# Fostering accessible online education using Galaxy as an e-learning platform

**DOI:** 10.1371/journal.pcbi.1008923

**Published:** 2021-05-13

**Authors:** Beatriz Serrano-Solano, Melanie C. Föll, Cristóbal Gallardo-Alba, Anika Erxleben, Helena Rasche, Saskia Hiltemann, Matthias Fahrner, Mark J. Dunning, Marcel H. Schulz, Beáta Scholtz, Dave Clements, Anton Nekrutenko, Bérénice Batut, Björn A. Grüning

**Affiliations:** 1 Bioinformatics Group, Department of Computer Science, Albert-Ludwigs-University Freiburg, Freiburg, Germany; 2 Khoury College of Computer Sciences, Northeastern University, Boston, Massachusetts, United States of America; 3 Institute for Surgical Pathology, Medical Center, University of Freiburg, Freiburg, Germany; 4 Faculty of Medicine, University of Freiburg, Freiburg, Germany; 5 Avans Hogeschool, Breda, the Netherlands; 6 Erasmus Medical Center, Clinical Bioinformatics Group, Department of Pathology, Rotterdam, the Netherlands; 7 Faculty of Biology, University of Freiburg, Freiburg, Germany; 8 Spemann Graduate School of Biology and Medicine, University of Freiburg, Freiburg, Germany; 9 Faculty of Medicine, Dentistry and Health, University of Sheffield, Sheffield, United Kingdom; 10 Institute for Cardiovascular Regeneration, Goethe University, Frankfurt am Main, Germany; 11 University of Debrecen, Faculty of Medicine, Dept. of Biochemistry and Molecular Biology, Debrecen, Hungary; 12 Johns Hopkins University, Baltimore Maryland, United States of America; 13 Center for Comparative Genomics and Bioinformatics, Penn State University, State College, Pennsylvania, United States of America; SIB Swiss Institute of Bioinformatics, SWITZERLAND

## Abstract

The COVID-19 pandemic is shifting teaching to an online setting all over the world. The Galaxy framework facilitates the online learning process and makes it accessible by providing a library of high-quality community-curated training materials, enabling easy access to data and tools, and facilitates sharing achievements and progress between students and instructors. By combining Galaxy with robust communication channels, effective instruction can be designed inclusively, regardless of the students’ environments.

## Introduction

The current pandemic bares the harsh reality that online education is here to stay and hence, universities need to face the challenge of adapting to a new paradigm in a very short period of time. Online teaching comprises a unique set of challenges, being the decline of motivation one of them [1]. The collective grief for a world that will never be as we knew it, makes the learning curve steeper for both trainers and trainees. More empathy than usual is, therefore, required in assisting the learning process.

This situation is also rekindling the issue of the equity of opportunities to access education. Some education centres are alleviating the issue by lending equipment to their enrolled students that do not have access to those resources. Despite its drawbacks, online learning also provides us with many opportunities.

One of the greatest advantages of online education is its flexibility, a feature that makes education compatible with competing responsibilities, such as homeschooling and demanding work schedules or students coping with distracting environments. Learner isolation and loneliness are two additional issues responsible for learner failure or lack of satisfaction in online learning experiences [2]. The creation of effective online learning communities is critical for addressing these effects on the learning process. Online learning communities aim to create personal interactions that happen naturally in a live setting. Social constructivism theory holds that setting up a learning community is an essential element for the construction of knowledge [3,4].

As an attempt to overcome the situation, virtual teaching platforms are replacing traditional training scenarios during this pandemic. Video calls, chats, and shared documents are presented as the combination of tools for a successful online teaching activity [5]. However, the proliferation of tools that students are not familiar with adds cognitive load to the learning process [6]. Moreover, although some level of redundancy in communication channels is useful in case of failure, having different simultaneous ways to interact can be confusing for the students.

In this work, we present Galaxy as a teaching platform that addresses the recommendations and features required for effective learning [7], such as sharing capabilities, formative assessment of the progress, and checking exams to keep the students engaged. The Galaxy Training Network (GTN) [8] provides more than 150 hands-on training materials—created by the community—covering realistic use cases on small but meaningful data for many scientific disciplines from classical omics to imaging, ecology, or climate analyses. All the tutorials are accessible to everyone, without any cost, and so are the public Galaxy servers that provide the tools and computational infrastructure to run trainings. More importantly, the cloud infrastructure is accessible from a web browser with no need for special hardware specifications or extra installation of software.

## Galaxy platforms and training infrastructure as a service

Galaxy is an open, web-based platform for accessible, reproducible, and transparent computational biological research. Galaxy offers thousands of tools ranging from classical omics, statistics, and machine learning, to imaging, ecology, and climate software. All tools and Galaxy features are accessible via API as well as a graphical user interface. Galaxy has been used by more than 100,000 life science and medical researchers, many without programming knowledge. The web-based nature of Galaxy helps enormously with accessibility since no specific hardware or software requirements are needed to run an analysis.

More than 125 public Galaxy servers around the world (https://galaxyproject.org/use/) provide free access to different tool kits and computational resources. A High-Performance Computing (HPC) environment runs the tools launched by the users in the backend with the specified data. In order to avoid potential overload in the HPC cluster, a special queue is provided for the training activities scheduled through the Training Infrastructure as a Service (TIaaS, https://galaxyproject.eu/tiaas) [9]. This service is completely free and unrestricted for any Galaxy tool, including all training tutorials available in the GTN [8]. The most prominent Galaxy instances, such as the three main public usegalaxy*. servers already have all the tools necessary for the GTN as well as the training data available locally. These instances come along with thousands of CPU and terabytes of RAM and therefore allow running several parallel trainings without hindering regular Galaxy users. For example, more than 150 training sessions with more than 4,500 trainees were executed on the European Galaxy server since mid-2018, while more than 23,000 regular users were not hindered in running their data as normally [9]. All Galaxy servers share the same setup; therefore, if trainees need more specialised software that was not available on the Galaxy server used for the training, they can easily switch to another server. In addition to scheduling training runs, TIaaS enables the lecturers to assess the progress of the students in real time in a General Data Protection Regulation (GDPR)-compliant way [9].

The combination of Galaxy and the GTN materials occupies a complementary space to projects such as edX (https://www.edx.org/), Coursera (https://www.coursera.org/), and Rosalind [10]. While these platforms allow the development of the conceptual and methodological dimensions of bioinformatics education, their capacity to transmit the necessary skills for the adequate domain of bioinformatics computational tools is limited [11]. Setting up an adequate environment for effective bioinformatic analysis can be a challenge, especially for students who lack technical knowledge, for which Galaxy is an ideal solution. Rosalind is perhaps the closest match. Like Galaxy + GTN, Rosalind also includes an integrated analysis platform and is focused on bioinformatics. Unlike Rosalind, Galaxy can also be used to do actual research. Rosalind does have very clearly defined learning paths [10], while support for these in GTN is in early stages. Like Galaxy, edX and Coursera have a global community of contributors and are easily expanded to cover new topics.

## Galaxy encourages sharing as a fundamental concept of open science

Teaching with Galaxy is an opportunity to communicate the principles of open science and reproducibility [12,13]. Galaxy provides features that make sharing of data, complete analysis histories, and reusable workflows particularly effortless. When not restricted by ethical considerations, all data and analysis steps can be easily made available with complete versioning information and parameter settings.

### Data sharing in Galaxy

Students can upload their own datasets that will live in their Galaxy workspace, called history in Galaxy. The simplest way to do it is via the web front-end, although alternative mechanisms are available for larger datasets (https://galaxyproject.org/tutorials/upload/). For teaching purposes and to reduce processing times, the GTN data are usually small, still yielding meaningful results for its scientific interpretation. Alternatively, all GTN datasets are stored and shared via the Galaxy Data Libraries (https://galaxyproject.org/data-libraries/) on the main, public usegalaxy.* servers (https://github.com/usegalaxy-eu/shared-data/). The power of this feature resides in its scalability, allowing data versioning and preventing data duplication across different user Galaxy histories and long waiting times when uploading datasets into Galaxy. To optimally benefit from this feature, trainers can prepare the Data Libraries in advance.

Galaxy extracts the metadata associated with every dataset, including the file format, a crucial element in life sciences data analysis. Although there are many different formats available, students need to learn, at least, how to identify, convert, and visualise the most popular ones. Based on our experience, reinforcing the basic concepts and the different file formats is relevant for the students: first, to understand which information is included in which file format; and second, to acquire the scientific vocabulary for future competent communication with colleagues.

### Analysis histories sharing in Galaxy

Galaxy histories are the central feature to ensure data analysis transparency, reproducibility, and shareability. Every data analysis step (tool version, selected parameters, etc.) along with the entire provenance of a dataset are captured in Galaxy histories and constantly available for the students.

In an online teaching environment, providing students with real-time feedback can be challenging, due to the lack of visual contact and time shift restrictions. Consequently, a straightforward shareability method becomes extremely important for the progress assessment and final evaluation of the goals achieved.

Galaxy provides a set of features to facilitate shareability, either with only one user (e.g., between peers or with the instructor), with defined groups or publicly.

### Workflow sharing in Galaxy

Tools can be linked together to perform multistep and arbitrarily complex analyses. In Galaxy, these multistep processes can be saved as reusable workflows. Workflows can then be run on different input datasets. Workflows, like histories, can also be shared with individuals, groups, or published to everyone on the server. If a given tool yields an error during the process, the workflow is paused and the successive step would not be executed. In a teaching context, workflows are particularly useful to run a particular analysis that can be exported and shared with the students or the supervisors.

## Proposed teaching format within the Galaxy framework

Trainers can bring all the Galaxy features together to create effective e-learning activities. Among the types of online learning, Galaxy and the GTN training materials are suitable either for online courses—defined as the delivery of training material asynchronously for students to learn at their own pace—or for live virtual training, in which the trainers and the students meet in a virtual meeting room simultaneously.

Web-based online teaching based on well-elaborated material and a scalable infrastructure need communication channels to boost the interaction among the participants. It is the trainers’ responsibility to establish adequate communication channels to engage students and to create a respectful environment in which students feel empowered to share their knowledge and learn from each other [4,14]. In this sense, students must be guided through the available technology by clearly stating which particular tools will be used and in which way.

**Fig 1:**
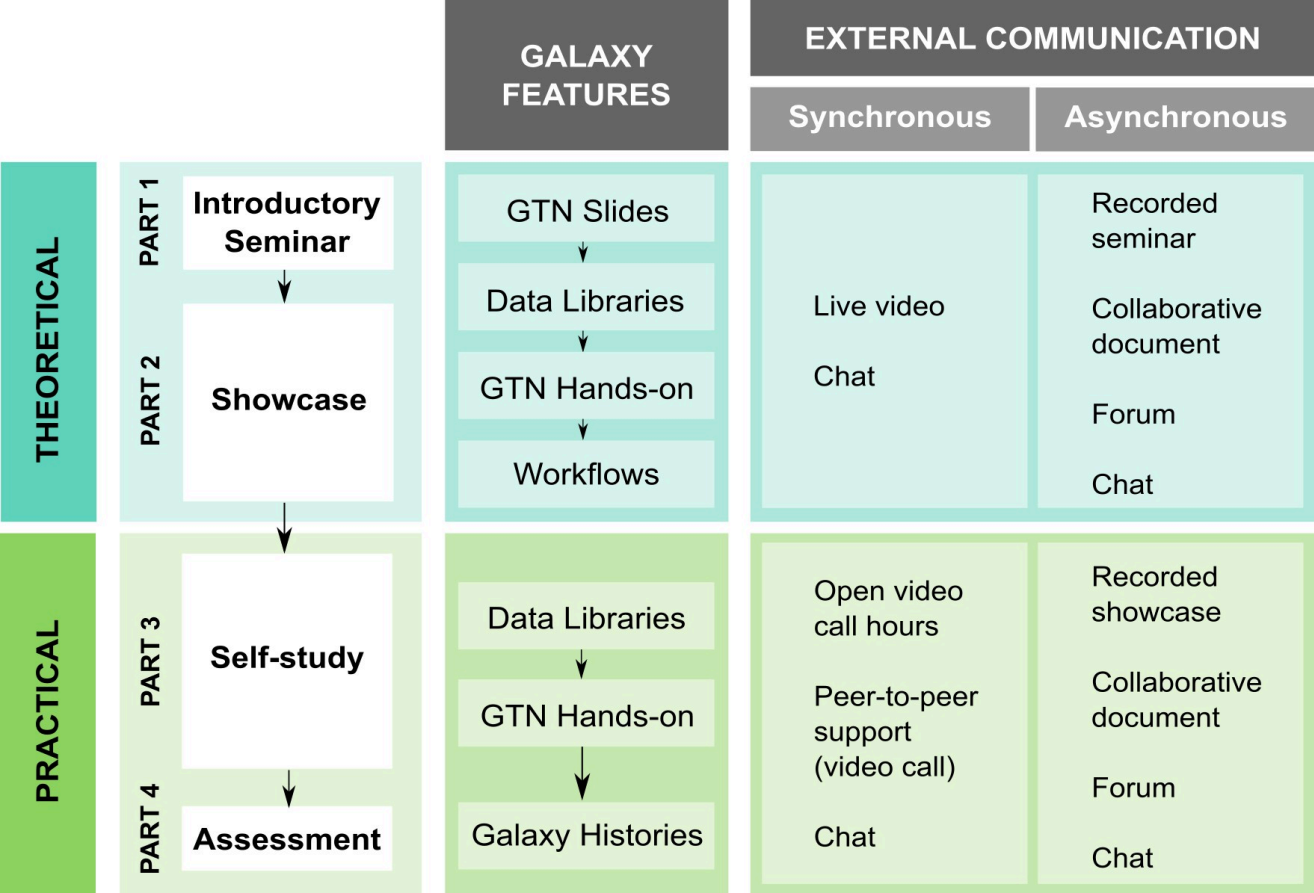
Proposed teaching format including theoretical using Galaxy features and external communication channels.

The effect of learner control on the pace of the lesson improves their engagement and satisfaction [15], so a combination of synchronous and asynchronous communication channels need to be jointly considered during the preparation phase of the training (Fig 1). Asynchronous communication allows a higher degree of self-management on the side of the students [16]. Furthermore, it has been found that it is more useful for task-oriented communication and to facilitate high levels of thinking, such as critical thinking [17,18]. Synchronous communication, on the other hand, has been shown to be more effective in promoting social interactions [19].

Video calls can be classified as synchronous channels. The basic features are screen sharing, chat, and recording—with the consent of the participants for GDPR compliance. Whiteboards, polling, and remote control rights can increase participation. Although many commercial tools (e.g., Google Meet, Zoom, Microsoft Teams) offer plenty of these features, open-source resources cover the most important aspects too (e.g., BigBlueButton, Jitsi). It is important to highlight here that some of these options require the installation of additional software and/or creation of email accounts with different providers, which could limit the freedom of choice of the students.

One consideration to guarantee inclusiveness is to bear in mind that the synchronous channels provide interactivity only for those students that are able to join the live sessions. Time zone shifts, distracting environments, and other limitations might prevent the attendance of part of the student body. That makes the asynchronous channels especially convenient: Regular exchange of emails, posts in forums, and shared documents can be used to provide integrated feedback, comments, or suggestions and to ask questions in an asynchronous way, which can benefit the less active students. Chats serve as synchronous communication channel for those attending the live sessions as well as for those watching the recordings in an asynchronous way.

Based on the above ideas, we suggest four parts for an e-learning approach with Galaxy.

### First part: Introductory seminar

To provide students with some context on the scientific background of the training course, a short presentation (30 to 60 minutes) as a general introduction is recommended (Fig 1). However, just replicating an online version of a classroom lecture lacks its strengths and pedagogical characteristics [20]. Thus, the materials need to be adapted and the interactivity will have to be promoted in order to increase the engagement of the students [21].

For accessibility and equity of opportunities, the presentation should be recorded (e.g., with *Open Broadcaster Software*) and shared with the students that cannot attend the live session. Ideally, the slides—the GTN offers introductory slides as well—will be shared with the students in a printable format with enough time ahead of the course.

During this session, questions will inevitably arise. A possible way to address them is to ask the students to write them down—in the chat or a shared document—and answer them at the end of the presentation. Planning frequent breaks will make questions flow more thoroughly. The shared document with questions and answers can stay active for some time so that the students that are watching later can also formulate their questions in equal conditions.

### Second part: Showcase

After the presentation of the scientific topic, a live demonstration of the data analysis could take place (Fig 1). This demonstration is completely optional as the GTN materials are comprehensive enough to cover every aspect of the procedure. Nonetheless, a detailed demonstration with exhaustive commenting of every step can help ensure that the students can later repeat them autonomously. The recommendation for the lecturers is to use two monitors, one for the GTN material and another one for the Galaxy sessions shared with the students, always bearing in mind that the same assumption cannot be made on the students’ side.

### Third part: Self-study session

A flexible arrangement is imperative for e-learning. It cannot be assumed that every student can find the time for self-study during the day, thus a self-paced session with a few days to repeat the—ideally recorded—demonstration is recommended (Fig 1). All the GTN tutorials are designed as self-study material with clear objectives, detailed step-by-step guided instructions through the data analysis, sometimes even including supporting materials.

Learners need some time to get used to the interface of the tools and to explore the parameter space. According to the constructivist pedagogical approach, students construct their understanding through experiences and reflecting on them afterwards [19,20]. It is encouraged to artificially create short erroneous tasks that are not covered by the GTN materials, with the purpose of provoking errors that force students to hypothesise about the issue and find out solutions themselves rather than being guided [22]. Students would be active participants and no longer passive receptors: Reading the error messages and restarting tools with the appropriate parameters will improve their problem-solving and critical thinking skills. In this scenario, sharing Galaxy histories is again especially advantageous to debug, to request feedback, and to foster discussions with other students.

Even in this student-centred part, the role of the instructor should not be diminished. Teaching presence is relevant to guide students through the different stages of learning: exploration, integration, and application [23]. The different communication channels should, therefore, be open over the entire semester. Open video call hours, similarly to the open-door policies, are also a valuable mechanism to enable the discussions and encourage questions.

The suggested approach requires very little teaching personnel: During the theoretical live parts, questions can immediately be answered by the instructor. The self-study sessions are based on thoroughly designed and tested training material that allows for self-control and rarely creates problems. Thus, the few arising issues are scattered over time and can be addressed in the chat or shared document, or if more complicated by sharing the Galaxy history with the instructor.

### Fourth part: Assessment

The GTN tutorials are exemplary work packages that can be distributed to students as homework (Fig 1). To assess the level of understanding during the previous parts of the training, the GTN tutorial can be used with different and more complex datasets. The degree of complexity of the data can vary from real-world datasets or the data from the Personal Genome Project (https://www.personalgenomes.org/, https://galaxyproject.eu/posts/2020/01/16/pgp/). A more advanced exercise to promote critical thinking can be to hand over an open-access article which data analysis will have to be reproduced [19], which can be shaped as a group activity to simulate the in-person collective projects.

Once the homework has been performed, students can deliver the results, together with the steps in the analysis, and get feedback from the trainer or other students by sharing their Galaxy histories. Time stamps and metadata on every dataset are particularly useful to prove authorship. The role of the instructor is again vital at this stage to create a collaborative environment in which students can learn and help each other; in contrast to a model where the teacher is an authority on the subject [14]. Furthermore, to develop their scientific writing skills, the students can also write a report interpreting the results they obtained from the data analysis in a structured way.

After all the assessments have been collected, students may receive direct feedback on their analysis, and optionally, a gold-standard Galaxy history can be distributed as a reference.

## User stories

### Mark Dunning–The University of Sheffield, UK

Like many educational institutions around the world, we have been faced by the sudden prospect of having to convert our existing teaching offering to an online setting. A particular challenge within our faculty was that MSC students about to commence their final project work were no longer able to access the laboratories, meaning that alternative “dry-lab” projects had to be allocated. This created a large cohort of students with little or no computational experience suddenly having to take a crash course in Bioinformatics. The resources provided by Galaxy and GTN was an ideal solution to address this need.

Requiring learners to install Bioinformatics and command-line software on their own devices prior to a course can be problematic at the best of times but is obviously exacerbated by demonstrators not being able to physically access the learners’ device. With the Galaxy platform being available online, this is no longer a concern. Moreover, the learners can feel confident that they will be able to access the same tools on their own after the workshop. Although the tools are presented in a friendly online interface, we reassure the learners that the tools they are using are the same ones we would use as practicing Bioinformaticians as part of a best-practice workflow.

Aside from the tools themselves, having access to detailed and clear notes is essential during an online workshop. When the lead instructor in a workshop is relying on their home broadband connection, some audio and video drop-out can be expected. However, if this occurs, the learners are able to keep up to speed by following the notes. They also greatly benefit from materials (both written and recorded) being accessible after the workshop so they can review at their own convenience.

### Helena Rasche and Saskia Hiltemann–Erasmus Medical Center, Rotterdam, The Netherlands

After significant experience training individual classrooms of students in Europe and beyond, often flying to different countries to teach, we and several of our colleagues formed the Gallantries project to teach basic bioinformatics skills remotely. We pioneered a hybrid training approach wherein we broadcast a training event to multiple classrooms of students, a sort of bridging step to the current reality of completely remote teaching. We ran two pilot workshops during a Mozilla mini-grant to prove the feasibility of a hybrid teaching approach, using the GTN materials with success.

For this, we leveraged Galaxy as a training platform. It provided an ideal e-learning environment for our students, as all remote learners could access the same website for their bioinformatics learning. Everyone worked within the same environment, all steps could be tested ahead of time and were known to be reproducible. Using Galaxy and the GTN let us remove a huge source of variability in our training via standardised environment and tested training materials. Having the training materials designed from the start to support self-study was likewise a boon. In the case of broadcast issues, students could still follow along with the materials on their own.

Another benefit of having everyone centralised on a single Galaxy server was that we could share histories with students, either as examples of correct work or as a way to help students catch up to the instructor when they fell behind.

The TIaaS dashboard was additionally created in direct response to these hybrid training events, as teachers needed significantly more visibility into student progress to run efficient workshops. TIaaS dashboard takes advantage of students using a central server to report their progress, enabling our teachers to quickly move through portions of the lessons with which students do not struggle.

### Melanie Föll and Matthias Fahrner-University of Freiburg, Germany

As in previous years, we taught a Galaxy proteomic data analysis course for Molecular Medicine master students. Due to the given COVID-19 situation, we had to transform the course into an online format. We were able to quickly adapt to those exceptional circumstances, thanks to the great infrastructure that Galaxy and GTN provide. We used the Jitsi video call platform for the course which was kindly provided by the University of Freiburg. The course consisted of multiple meetings covering diverse proteomic data analysis topics. Each meeting started with a theoretical introduction lecture, followed by a live demonstration of the analysis in Galaxy and time for questions and discussions. For improved learning experience, the students were asked to repeat the analysis by following the GTN material as homework. As an additional motivation, certificates were provided for students who submit their Galaxy histories of the GTN tutorials. The shared Galaxy histories also helped during troubleshooting and to address students’ questions more effectively. Interestingly, fewer errors occurred than in offline courses probably because students could follow the training at their own pace. We were able to transmit all course contents effectively and in a straightforward manner due to the great infrastructure of Galaxy and the GTN. The high teaching capability and potential using Galaxy and GTN tutorials are also reflected in the unanimously positive feedback which we received from the students.

### Marcel Schulz–Goethe University Frankfurt am Main, Germany

Due to the COVID-19 pandemic, we had to turn the computational epigenomics course for Master Bioinformatics students into an online course. We designed the course with recorded online lectures in which the theoretical knowledge about epigenomics, the common analysis tools and their statistical principles were explained. The videos could be watched at any time, such that the students worked on their own pace. Galaxy was used for the exercises that we gave the students because it has many different tools installed and ready for use. It was particularly important to us that the students would not have to deal with installation issues, which would have been difficult and time consuming for us to help fix, as we wanted to cover a large set of tools and different data types and file formats. Further, because people were working mostly from home, it was not clear to us whether people could connect remotely to computers that would be able to run the time-consuming analyses.

Because the students would solve the assignments and have the resulting data in their histories, it was easy to conduct integrative analyses in later assignments, where the students would reuse their own analyses results from initial data processing steps, such as read alignment and peak calling. For evaluation, the simple history sharing functionality in Galaxy made it easy for us to check whether students were able to solve the tasks correctly. In addition, we had them answer a few questions regarding the data they had produced to provoke critical thinking about the used tools and the results of the data analysis.

A mandatory weekly video call was used to help answer questions about the lectures, in addition to a chat channel that we constantly monitored to solve pressing issues. On the call, we discussed results from the assignments using an interactive approach, where one of the students, informed ahead of time, would present his/her results for one part of the assignment. This sometimes led to an active discussion among peers and students, which was very enjoyable, and resolved misconceptions.

## Conclusions

Here, we present Galaxy as a teaching platform in response to the recent changes in the education needs due to the COVID-19 world pandemic. Galaxy provides a wide variety of ready-to-use features that facilitate the online learning process. Those features, together with the free materials available at the GTN, can be combined for an integrative teaching design that makes education accessible for many different student profiles.

In terms of addressing the content, an effective format seems to be a combination of a webinar to introduce the main theoretical topic together with practical self-study sessions in which students can follow very detailed training materials. Ideally, the first part could be recorded for flexibility purposes, always with the consent of the presenters to be GDPR compliant. If a demo is shown instead of—or in addition to—a webinar, all the steps need to be very slowly explained and the recordings help in this case especially if another software needs to be shown side by side with the video. The practical part requires the training materials to be more detailed with the advantage of bringing flexibility to the students and addressing everyone’ needs.

The uncertainty and complexity of the current pandemic situation complicate the prediction of future trends in education, but clearly, adapting to an e-learning setting has many benefits. Our hope is that the current global situation will make us reflect on the accessibility of education in equal terms for every underprivileged community and, more importantly, act upon it.
